# Aflatoxin exposure among lactating women in southern Ethiopia

**DOI:** 10.1002/fsn3.1968

**Published:** 2020-10-26

**Authors:** Bergene Boshe, Samson Gebremedhin, Fikadu Alemayehu, Mesfin Eshete, Mestawet Taye, Barbara J. Stoecker

**Affiliations:** ^1^ School of Nutrition, Food Science and Technology Hawassa University Hawassa Ethiopia; ^2^ School of Public Health Addis Ababa University Addis Ababa Ethiopia; ^3^ School of Animal and Range Sciences Hawassa University Hawassa Ethiopia; ^4^ College of Human Sciences Oklahoma State University Stillwater OK USA

**Keywords:** Aflatoxin B_1_, agroecology, food, lactating women, seasonality, urinary aflatoxin M_1_

## Abstract

In Ethiopia and many other low‐income countries, little is known about the exposure of lactating women to aflatoxin, which is a major health concern to the mother and her nursing infant. We determined the aflatoxin B_1_ contamination of family foods (AFB_1_) and urinary aflatoxin M_1_ (AFM_1_) of lactating women in Sidama, southern Ethiopia, and compared the levels across agroecological settings (lowland, midland, highland) and two seasons. We conducted two surveys (*n* = 360) that represented the dry and wet seasons of the locality. AFM_1_ and AFB_1_ were determined using enzyme‐linked immunosorbent assay (ELISA). Statistical analysis was made using Mann–Whitney *U* test and Kruskal–Wallis test. The median (interquartile range) AFB_1_ was 0.94 (0.63–1.58) ppb. AFB_1_ was detected in 95.6% of the food samples, and 13.6% exceeded the 2.0 ppb threshold. We observed an increasing trend for aflatoxin exposure from highland to lowland (*p* < .001), but there was no difference between seasons (*p* = .743). The median (interquartile range) urinary AFM_1_ was 214 (undetectable to 2,582) ppt, and AFM_1_ was detectable in 53.3% of the samples. Urinary AFM_1_ showed significant difference among agroecological zones (*p* < .001) but not between seasons (*p* = .275). A significant but weak correlation was observed between AFB_1_ and urinary AFM_1_ (*r_s_* = 0.177, *p* = .001). We concluded that lactating women in Sidama, especially those in the lowland area, have unsafe exposure to aflatoxin.

## Graphical abstract

Aflatoxin B1 was detected in nearly all of the food samples, and in 14% of the cases, it exceeded the limit of 2.0 ppb. Aflatoxin M1 was detected in nearly half of the urinary samples. Increasing trend for aflatoxin exposure from highland to lowland was observed.

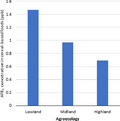

## 1 BACKGROUND

Aflatoxins are a group of highly toxic fungal food contaminates with serious health consequences (Payne & Brown, 1998). Aflatoxin contamination of food is a major risk factor for hepatocellular carcinoma, and it explains 5%–28% of the global burden of hepatic cancer (Liu & Wu, 2010). Exposure to unsafe levels of aflatoxin during early life, including in utero exposure and exposure through breast milk and complementary foods, may lead to growth impairment, immune suppression, and micronutrient deficiencies (Wild, 2017; Williams et al., 2004). From an economic standpoint, market losses secondary to contamination of food and feed with aflatoxins and human capital losses due to aflatoxicosis annually costs the global economy billions of dollars (Wu & Khlangwiset, 2010).

Several types of aflatoxins have been identified so far; however, four types (B_1_, B_2_, G_1_, G_2_) and two secondary metabolites (M_1_, M_2_) are significant food contaminants (Williams et al., 2004). Especially, aflatoxin B_1_ (AFB_1_) is regarded as the most potent carcinogen. In humans and animals exposed to unsafe levels of AFB_1_, the toxin metabolizes to aflatoxin M_1_ (AFM_1_), and is secreted into milk and excreted via urine and feces (Marchese et al., 2018). Aflatoxin exposure of humans and animals can be monitored using multiple indices including measurement of contamination of foods, plasma concentration of aflatoxin–albumin adducts, and excretion of hydroxylated metabolites in urine and breast milk. Urinary aflatoxin excretion is considered as a simple index of recent exposure (WHO, 2018).

Studies from low‐ and middle‐income countries suggested that exposure of lactating women to unsafe levels of aflatoxin is common. Study from western Iran reported that AFM_1_ was found in all human breast milk samples tested (Maleki et al., 2015). Similarly, in Nigeria, 95% of women had detectable urinary AFM_1_ (Alegbe et al., 2017). Exposure of infants to AFM_1_ via breast milk may cause stunting and growth faltering (Magoha et al., 2014; Sadeghi et al., 2019). A study in Tanzania reported a statistically significant negative relationship between infants’ anthropometry and AFM_1_ exposure through breastfeeding (Magoha et al., 2014).

Studies suggested that the epidemiology of aflatoxin contamination of the food system is liable seasonal and agroecological variations (Elaridi et al., 2017; Kılıç Altun et al., 2017). Aflatoxin contamination tends frequently to occur in warm and humid settings (Wild, 2017), and various seasonal patterns have also been reported (Elaridi et al., 2017; Kılıç Altun et al., 2017). Accordingly, we analyzed the AFB1 contamination of family foods and urinary excretion of AFM_1_ among lactating women in Sidama, southern Ethiopia, and compared the levels across different agroecological settings (lowland, midland, and highland) and seasons (wet and dry).

## 2 MATERIALS AND METHODS

### 2.1 Study design and setting

We collected data in August 2017 (wet season) and the March 2018 (dry season) in Sidama zone, through two independent cross‐sectional surveys. The actual surveys were carried out in Hawassa Zuria, Dale, and Hula districts, representing the lowland (<1,750 m above sea level (a.s.l)), midland (1,750–2,300 m a.s.l), and highland (>2,300 m a.s.l) agroecological zones of Sidama. Sidama zone covers nearly 10,000 km^2^ area and has diverse climatic conditions with altitude ranging from 1,200 m to 2,800 m a.s.l. Depending on the agroecological feature, the mean annual rainfall ranges from 400 to 1,600 mm and average annual temperature varies between 15 and 25°C. In 2017, the zone had nearly four million inhabitants of whom 95% were rural dwellers (Population Census Commission, 2008). The economy in the area is dependent on rainfed subsistence farming, and major crops grown are maize, *enset* (*Enset ventricosum*), and coffee. *Enset* is the main staple throughout Sidama while maize is so in the lowlands (UNDP, 2002).

### 2.2 Study participants

All lactating women nursing infants and young children 6–23 months of age were considered eligible for the study. Women nursing infants 0–5 months were excluded because the larger project from which this study originated was designed to evaluate the aflatoxin contamination of complementary foods prepared for children 6–23 months. In the second‐round survey, study participants who took part in the first survey were excluded with the concern that information received in the first round may affect the findings of the latter survey.

### 2.3 Sample size determination and sampling procedure

Priori sample size was determined using G*Power 3.1 program (Faul et al., 2007), assuming that aflatoxin concentration in food and urine will be compared between seasons and among agroecological settings using independent *t* test and one‐way analysis of variance, respectively. Ultimately, assuming 95% confidence level and 80% power, a sample size of 180 subjects per survey round, was deemed adequate to detect a medium effect size of 0.3 (Sullivan & Feinn, 2012).

In both of the seasonal surveys, the study participants were selected using a multistage cluster sampling technique. Initially from the 19 administrative districts of Sidama, Hawassa Zuria, Dale, and Hula districts were selected to represent the aforementioned three agroecological settings. Then from each district, two villages with the required agroecological feature were chosen, and from each village, 30 lactating women were recruited using lottery method. When a selected woman failed to take part in the study for any reason, we have replaced her with an eligible woman from an adjacent household.

### 2.4 Measurement and data collection procedures

Socio‐demographic information and knowledge and practice of the participants toward mold contamination of foods were assessed using a questionnaire prepared in the local language. The tool was pretested and administered by trained enumerators. Maternal anthropometric measurements (height and weight) were taken following standard procedures using calibrated instruments. During the interview visits, 40 ml of morning midstream urine and 100 g of cooked cereals‐based foods (mainly maize‐based foods and few made up of wheat and teff) and *Enset* products prepared for household consumption were collected in clean plastic containers and were properly labeled. Food and urine samples then were transported to the Nutrition and Food Science Laboratory of Hawassa University in an icebox and kept frozen at −20°C until analyzed.

### 2.5 Laboratory analysis

AFB_1_ and urinary AFM_1_ concentrations were determined using enzyme‐linked immunosorbent assay (ELISA) kits (Helica Biosystems Inc., Santa Ana, California, USA). Urine samples were centrifuged at 3,000 rpm for 10 min to remove precipitate, and standards of 0, 150, 400, 800, 1,500, and 4,000 pg/ml were prepared. Samples were diluted 1:20 ratio using distilled water, and the assay protocol provided by the manufacturer was followed to determine urinary AFM_1_ (Helica Biosystems, 2020a). Similarly, for AFB_1_ analysis, 20 g of the food samples was first ground to fine particle size (50% passed through a 20‐mesh screen) and 100 ml of solvent (70% methanol and 30% distilled water) added. After shaking the solution for 5 min, 10 ml of sample was filtered by Whatman qualitative filter paper grade 2 (125 mm diameter) for testing. Ultimately AFB_1_ level was determined following standard procedures recommended by Helica Biosystems Inc (Helica Biosystems, 2020b). The limits of detection (LoD) for AFB_1_ and AFM_1_ tests determined by the manufacturer were 0.2 ppb and 0.15 ppt respectively. Recovery data were 96.4 for AFB_1_ and 96.5% for AFM_1_, respectively. For both tests, the coefficient of variation was below 10%.

### 2.6 Data management and analyses

We used SPSS 24 for data analysis. Normality of AFB_1_ and AFM_1_ was visually assessed using histograms and tested via Kolmogorov–Smirnov statistic, and both indices demonstrated right‐skewed distributions. Transformations were attempted but failed to normalize the distributions. Accordingly, AFB_1_ and AFM_1_ were described using median and interquartile range (IQR) and compared among classes of agroecology and seasons using nonparametric tests. Mean AFB_1_ and AFM_1_ ranks were compared between seasons and across agroecological zones using Mann–Whitney *U* test and Kruskal–Wallis test, respectively. For Kruskal–Wallis test with global statistical significance, pairwise post hoc tests were performed. For AFB_1_, the proportion of food samples that exceeded the European Union (EU) thresholds of 2.0 parts per billion (ppb) was calculated (European Union, 2006). Correlation between AFB_1_ from food samples and urinary AFM_1_ was measured using Spearman rank‐order coefficient (*r_s_*).

### 2.7 Ethical considerations

The protocol was reviewed and approved by the Institutional Review Board of the College of Medicine and Health Sciences, Hawassa University. Data were collected after securing informed consent from all study participants. The study was implemented in confirmation with national and international ethical standards for research involving human subjects including the Declaration of Helsinki.

## 3 RESULTS

### 3.1 Characteristics of the respondents

The basic characteristics of 360 lactating women interviewed for the study are presented in Table 1. The mean (±*SD*) age of the women was 26.3 (±4.4) years, and about one‐third were between 18 and 24 years of age. Nearly all (99.4%) were married, a quarter had no formal education, and 90% identified themselves as farmers or housewives. The median (IQR) monthly household income was 500 (400–800) Ethiopian birr (ETB), and four‐fifths earned less than 2,000 ETB per month (at the time of the survey, 1 USD was equivalent to 30 ETB). The median agricultural landholding size was 0.7 (0.4–1.3) hectares. Regarding anthropometric status, 78.1% of the women had normal body mass index.

**Table 1:** Basic characteristics of the respondents, Sidama zone, southern Ethiopia, August 2017 and March 2018, (*n* = 360)

Variables	Frequency	Percent
Agroecology		
Lowland	120	33.3
Midland	120	33.3
Highland	120	33.3
Age of mothers (in years)		
18–24	112	31.1
25–34	228	63.4
≥35	20	5.6
Educational status of respondents		
No formal education	100	27.8
Primary education	214	59.4
Secondary education or above	46	12.8
Occupation		
Farmer	197	54.7
Housewife	137	38.1
Petty trade	22	6.1
Others	4	1.2
Average monthly income (Ethiopian birr)		
1,000–1,999	283	79.1
2,000–2,999	49	13.7
3,000–3,999	10	2.8
4,000 or above	16	4.5
Agricultural land size		
No agricultural land	9	2.5
Less than 1 hectare	183	50.8
Greater than one hectare	168	46.7
Age of the index child (in months)		
6–11	174	48.4
12–17	104	28.9
18–23	82	22.7

### 3.2 Knowledge and practice toward aflatoxin contamination of food

Table 2 presents the knowledge and practice of the study participants toward mold contamination of food. Only 5.3% of the respondents were aware of aflatoxin. Most of the households dried their harvests prior to storage, and the popular approach was solar drying by spreading it on bare ground without any protection from soil contamination. In 51% of the households, grains were stored in traditional “*Gotera*” made up of wood, mud, and straw. Modern or improved stores were not used for a variety of reasons including lack of awareness and unaffordability. Nearly one‐third did not do anything to treat or disinfect their stores before stocking grains, and 18.9% reported that their stores were not dry and well ventilated.

**Table 2:** Knowledge and practice toward aflatoxin contamination of food Sidama zone, southern Ethiopia, August 2017 and March 2018, (*n* = 360)

Variables	Frequency	Percent
Practice of harvesting crop as soon as it is matured		
Yes	356	98.9
No	4	1.1
Practice of drying grains before storage		
Yes	336	93.3
No	24	6.7
Method of drying (*n* = 336)		
Sun drying on bare ground	166	49.4
Sun drying on plastic sheet	146	43.5
Indoor smoke drying	9	2.7
Others	15	4.5
Type of crop storage facility		
Traditional store “*Gotera*”	182	50.6
Nonplastic sacks	156	43.3
Plastic bag	10	2.8
Others (combination of the above)	12	3.3
Reasons for not using modern food storage facility		
Lack of awareness	144	40.0
Costly	138	38.3
Not locally available	44	12.2
Not important	34	9.4
Do you treat or disinfect the store before stocking grains?		
Yes	230	63.9
No	130	36.1
Do you think your store is dry and well ventilated?		
Yes	292	81.1
No	68	18.9
Before storage, do you sort out moldy grains?		
Yes	143	39.7
No	217	60.3
What do you do with the sorted out moldy grains? (*n* = 143)		
Feed to animals	73	51.0
Discard	59	41.3
Others	11	7.7
Use moldy cereals for brewing local beverages		
Yes	14	3.9
No	346	96.1
Do you think feeding animals with moldy feeds has any effect on human health?		
Has no effect	104	28.9
May affect human health	256	71.1
Heard about aflatoxin		
Yes	19	5.3
No	341	94.7

Only 39.7% of the women reported that they usually sort out moldy grains before storing grains for human consumption. Of them, 51% used the discarded moldy grains for feeding their domestic animals including chickens and milk cows. More than a quarter (28.9%) assumed that providing animals with moldy feeds does not have negative implications to human health. Smaller proportions (3.9%) used moldy cereals for brewing local beverages (Table 2).

### 3.3 Aflatoxin contamination of food

Of 360 food samples tested (mainly maize‐ and *enset*‐based foods and few made up of wheat and teff), the median (interquartile range) AFB_1_ was 0.94 (0.63–1.58) ppb. AFB_1_ was detected in 95.6% of the samples, and in 13.6% of the cases, the level exceeded the upper permissible level of 2.0 ppb set by the EU (European Union, 2006).

Table 3 compares the AFB_1_ levels between two seasons and across three agroecological settings. We observed no significant difference in AFB_1_ mean ranks between wet and dry seasons (*p* = .743). However, there was statistically significant difference among the three agroecological zones (*p* < .001) (Table 3). When performing post hoc comparisons, we detected significant differences between lowland‐midland (*p* = .014); midland–highland (*p* < .001); and highland–lowland (*p* < .001) pairs. Similarly, the proportion of samples that exceeded the upper EU permissible level were 30% and 10% in the lowland and midlands, respectively, while it was only 0.8% in the highland.

**Table 3:** AFB_1_ concentration of cereals‐based foods, Sidama zone, August 2017 and March 2018, (*n* = 360)

Factors	Positive samples Freq (%)	Median (IQR) (ppb)	*p‐*value	Min–max (ppb)
				
Lowland (*n* = 120)	117 (97.5)	1.47 (0.78–2.37)	<.001*	<LOD−4.01
Midland (*n* = 120)	117 (97.5)	0.97 (0.72–1.51)		<LOD−3.41
Highland (*n* = 120)	110 (91.7)	0.69 (0.45–1.09)		<LOD−2.53
Season				
Wet (*n* = 180)	177 (98.3)	0.94 (0.67–1.43)	.743	<LOD−3.90
Dry (*n* = 180)	167 (92.8)	0.95 (0.61–1.79)		<LOD−4.01

Abbreviation: LOD, limit of detection.

* Statistically significant difference was observed at 5% level of significance.

### 3.4 Urinary aflatoxin (AFM_1_) level

Among 360 urine samples tested, the median (interquartile range) urinary AFM_1_ was 214 (0–2582) ppt and AFM_1_ was detectable in 53.3% of the samples. There was significant difference in the AFM_1_ mean ranks across the agroecological settings (*p* < .001). Post hoc comparisons suggested significant differences between lowland–midland (*p* = .006); midland–highland (*p* < .001); and highland–lowland (*p* < .001) pairs. However, there was no significant difference between the two seasons (Table 4). Urinary AFM_1_ showed statistically significant but weak correlation with dietary AFB_1_ (*r_s_* = 0.177, *p* = .001).

**Table 4:** Urinary AFM_1_ among lactating women selected from three agroecological settings of Sidama zone, August 2017 and March 2018, (*n* = 360)

Factors (*n* = 360)	Positive samples Freq (%)	Median (IQR) (ppt)	*p‐*value	Min–max (ppt)
Agroecology				
Lowland (*n* = 120)	83 (69.2)	2,112 (<LOD‐5543)	<.001^*^	<LOD‐17243
Midland (*n* = 120)	71 (59.2)	477 (<LOD‐1846)		<LOD‐5225
Highland (*n* = 120)	38 (31.7)	<LOD (<LOD‐516)		<LOD‐5657
Season				
Wet (*n* = 180)	109 (60.6)	816 (<LOD‐2230)	.275	<LOD‐8857
Dry (*n* = 180)	83 (46.1)	<LOD (<LOD‐2705)		<LOD‐17243

Abbreviation: LOD, limit of detection.

* Statistically significant difference was observed at 5% level of significance.

## 4 DISCUSSION

The study confirmed that lactating women in Sidama zone, southern Ethiopia, have unsafe levels of aflatoxin exposure as measured by both AFB_1_ and urinary AFM_1_. There was an increasing trend for aflatoxin exposure from highland to lowland while there was no difference between seasons. The results further indicated that the knowledge and practice of the study participants and their households toward aflatoxin was suboptimal.

Almost all (96%) of the food samples had detectable AFB_1,_ and the level of contamination exceeded the threshold of 2.0 ppb in 13.6% of the samples. This is consistent to the understanding that the food system in Ethiopia is heavily contaminated with aflatoxin. A study in Gedeo zone, southern Ethiopia, that tested 150 maize samples from various sources including local markets and flour mills found that all the samples were contaminated beyond the safety level set by the EU (Chauhan et al., 2016). According to Worku and his colleagues, aflatoxin was detected in all stored maize samples collected from three regionals states of Ethiopia (Worku et al., 2019). Ayalew et al. (2006) detected AFB_1_ in 8.8% of the barley, sorghum, *teff,* and wheat samples collected from different parts of Ethiopia (Ayalew et al., 2006).

Urinary AFM_1_ was detected in 53.3% of the lactating women suggesting high levels of recent aflatoxin exposure. Alegbe and his colleagues in Yobe State, Nigeria, reported that 93% of the lactating women excreted AFM_1_ in their urine and 82% had AFM_1_ in their breast milk (Alegbe et al., 2017). Similarly, urinary AFM_1_ was detected in 84% of pregnant women from China (Lei et al., 2013). Most of the existing studies evaluated the aflatoxin exposure of lactating women using AFM_1_ concentration in breast milk. Studies conducted in Nigeria (Anthony et al., 2016) and Tanzania (Fakhri et al., 2019) reported that 90% and 38% of the tested breast milk samples exceeded the EU limit of 0.025 ppb. In Sudan, half of the breast milk samples were highly contaminated with AFM_1_ (Elzupir et al., 2012).

We found that the knowledge and the practice of the study participants and their households toward aflatoxin was suboptimal. Very small proportions were aware of aflatoxin and many harvest and store grains in ways that support proliferation of aflatoxin. Previous studies in Ethiopia also concluded the same. A study by Beyene and his colleagues in Amhara, Tigray, Oromia, and southern regions of Ethiopia reported that mothers caring for infants and young children had poor knowledge and practice regarding storage and processing of food and the consequences of aflatoxin exposure to humans (Beyene et al., 2016). A study in Wolaita zone of Ethiopia suggested only a very small proportion of farms were aware of aflatoxin and its consequences (Kibret et al., 2019).

Overall, there was an increasing trend for aflatoxin exposure from highland to lowland. This is compatible with the understanding that aflatoxin proliferation is favored by hot climate and the same had been documented by studies conducted in other sub‐Saharan Africa countries. A study in Zambia reported that maize and groundnut samples tend to be more frequently contaminated in the lowlands as compared to the other climatic zones (Kachapulula et al., 2017). In Kenya, contamination of multiple staples including maize was lower in temperate areas than in humid and semiarid zones (Sirma et al., 2016). The agroecological variations that we observed in Sidama can also be partly explained by the fact that maize is an important staple in the lowlands but not in the highland areas (UNDP, 2002). Studies suggest that maize is more frequently contaminated by aflatoxin than other staples (Mahato et al., 2019).

Urinary AFM_1_ is a valid biomarker of recent AFB_1_ exposure, and a strong correlation had been documented between dietary aflatoxin exposure and urinary excretion (Turner, 2013; Zhu et al., 1987). Several factors may contribute to the weak correlation (*r_s_* = 0.177) that we observed between AFB_1_ and urinary AFM_1_. First, we only measured the AFB_1_ contamination of cereal‐based foods, not the total dietary aflatoxin exposures. Further, we did not adjust urinary AFM_1_ for creatinine; as a result, AFM_1_ variations can partly be explained by interindividual differences in urinary dilution. Parallel to our finding, a study among Tanzania children reported a moderate strength of association between AFB_1_ intake and urinary AFM_1_ (*r* = 0.442) (Chen et al., 2018). A study in Brazil among adults observed a modest but significant correlation (*r* = 0.45) between urinary AFM_1_ estimated dietary intake of total aflatoxins (Jager et al., 2016). Studies from Malaysia (Sulaiman et al., 2018) and Brazil (Romero et al., 2010) observed no association between urinary AFM_1_ and frequency of consumption of different food groups.

Methodological limitations of the study must be taken into consideration while interpreting the findings of the study. We only measured aflatoxin contamination of cereal‐based foods (including *enset*) and did not take exposure via other food groups into considerations. The comparison across seasons and agroecological settings can be confounded by extraneous factors including duration of food storage, as well as humidity and temperature levels during harvest, which were not adjusted in our analysis. Further, the use of nonparametric tests for comparing levels of aflatoxin exposure might have compromised the statistical power of the study. The fact that lactating women nursing infants under the age of 6 months were excluded might theoretically limit the generalizability of the study. Finally, due to the reasons that Ethiopia does not have well‐established aflatoxin standards, we compared our findings with standards set by the EU.

## 5 CONCLUSION

The study confirmed that lactating women in Sidama zone, southern Ethiopia, have unsafe levels of aflatoxin exposure as measured by both AFB_1_ and urinary AFM_1_, suggesting possible health concerns both to the women and to their nursing infants. Aflatoxin exposure was higher in the lowland than in the highland, but there were no seasonal differences. The study also showed that the knowledge and practice toward aflatoxin was suboptimal in the area.

## CONFLICT OF INTEREST

The authors declare no conflicts of interest. The sponsor had no role in the design, execution, interpretation, or writing of the study.
